# Changes in salivary oxytocin after inhalation of clary sage essential oil scent in term-pregnant women: a feasibility pilot study

**DOI:** 10.1186/s13104-017-3053-3

**Published:** 2017-12-08

**Authors:** Yuriko Tadokoro, Shigeko Horiuchi, Kaori Takahata, Takuya Shuo, Erika Sawano, Kazuyuki Shinohara

**Affiliations:** 1 St. Luke’s International University, 10-1 Akashicho, Chuo, Tokyo, 104-0044 Japan; 2 Graduate School of Nursing Science, St. Luke’s International University, 10-1 Akashicho, Chuo, Tokyo, 104-0044 Japan; 3 St. Luke’s Maternity Care Home, 24 Akashicho, Chuo, Tokyo, 104-0044 Japan; 4 Hokuriku University, 3 Ho, Kanagawamachi, Kanazawa, Ishikawa 920-1181 Japan; 5 Graduate School of Biomedical Sciences, Nagasaki University, 1-12-4 Sakamoto, Nagasaki, 852-8523 Japan

**Keywords:** Pregnant women, Induction of labor, Complementary and alternative medicine, Aromatherapy, Clary sage essential oil, Inhalation, Salivary oxytocin, Uterine contraction, Salivary cortisol, Feasibility study

## Abstract

**Objectives:**

This pilot study using a quasi-experimental design was conducted to evaluate the feasibility (i.e., limited efficacy, practicality, and acceptability) of our intervention protocol involving inhalation of the scent of clary sage essential oil by pregnant women and measurement of their preinhalation and postinhalation oxytocin levels.

**Results:**

Participants were women of singleton pregnancies between 38 and 40 gestation weeks (N = 11). The *experiment group* (n = 5) inhaled the scent of clary sage essential oil diluted 50-fold with 10 mL of odorless propylene glycol for 20 min. Regarding *limited efficacy*, the oxytocin level 15 min postinhalation increased in 3 women and was unmeasurable in 2. The *control group* (n = 6) inhaled similarly without the 50-fold dilution of clary sage essential oil. Their oxytocin level increased in 2 women, decreased in 2, and was unmeasurable in 2. Uterine contraction was not observed in both groups. Regarding *practicality*, 3 of the 11 women could not collect sufficient saliva. The cortisol level decreased in both groups postinhalation. The protocol had no negative effects. Regarding *acceptability*, burden of the protocol was not observed.

*Trial registration* The Clinical Trials Registry of University Hospital Medical Information Network in Japan—UMIN000017830. Registered:  June 8, 2015

**Electronic supplementary material:**

The online version of this article (10.1186/s13104-017-3053-3) contains supplementary material, which is available to authorized users.

## Introduction

Medical induction of labor has been widely used [1–3]. However, it occasionally interferes with physiologic childbirth, necessitating the evaluation of the effects of complementary and alternative medicine for stimulating labor [4, 5]. Aromatherapy has been complementarily used for stimulating and strengthening labor contraction (e.g., repeated use of the scent of clary sage essential oil) [6–10]. However, there are few studies that have investigated the effects of aromatherapy on labor stimulation.

Labor involves uterine contractions (UCs) caused by oxytocin [11]. Clary sage essential oil is thought to stimulate labor by increasing the oxytocin level. However, studies evaluating changes in the oxytocin level of pregnant women by any intervention remain scarce. Thus, we conducted this feasibility study as a basis for future larger studies [12, 13].

This research was a pilot study to determine the feasibility of our intervention protocol in which pregnant women inhaled the scent of clary sage essential oil. Our primary objective was to evaluate the protocol’s limited efficacy in terms of the oxytocin level and UC. Our secondary objective was to evaluate the protocol’s practicality (i.e., saliva collection ability, cortisol level, and negative effects on participants) and acceptability (i.e., burden of intervention and scent perception).

## Main text

### Methods

This research was a feasibility pilot study conducted using a quasi-experimental design with two arms: experiment group and control group.

#### Participants and setting

The inclusion criteria included low-risk pregnant women between 38 and 40 gestation weeks before labor onset for planning spontaneous delivery. The exclusion criteria included age (< 25 or > 35) and aromatherapy allergy. The Additional file 1 shows details of participants’ criteria and request to participants until the intervention.

Sample size was not calculated because this pilot study aimed to provide a descriptive evaluation of the feasibility of the intervention protocol. Thus, about five participants for each group were considered appropriate. The first half of the participants was assigned to the control group and the last half to the experiment group. The feasibility pilot study was conducted at a birth center in Tokyo, Japan between June 2015 and August 2015.

The participants, experimental setting staff, and biologists measuring the outcomes were masked about the participant allocation.

#### Intervention

The inhalation intervention for each participant was started at 13:00. The participants rinsed their mouth with water, answered a questionnaire, and drank 100 mL of water. After 10 min from the water intake, the first saliva sample was collected [14] and the inhalation was started.

The experiment group inhaled the scent of clay sage essential oil (*Salvia sclarea*, Tree of life, Tokyo, Japan) [15] for 20 min. The scent was produced by bubbling using an air pump at 2.0 mL/min in an airtight bottle containing 10 mL of odorless propylene glycol diluted with 50-fold of clary sage essential oil and delivered 10 cm away from the nostril [16]. The Additional file 2 provides details of the inhalation intervention.

The control group inhaled similarly using the same device but without the dilution of clary sage essential oil with 10 mL of propylene glycol.

#### Outcome measures

For the primary objective, salivary oxytocin level was measured at four time points: 10 min preinhalation (baseline), and 15, 30, and 60 min postinhalation [17–21]. At each measurement point, at least 1.0 mL of saliva was collected in a polypropylene tube by passive drool after pooling saliva in the mouth for 3 min [22, 23]. When the volume was less than 1.0 mL, saliva was recollected as in the first attempt. The participants drank 100 mL of water again immediately after saliva collection at 30 min postinhalation. Salivary samples were frozen in a box containing dry ice after each collection point, and were then stored at − 80 °C following completion of each intervention.

Oxytocin level was assayed in duplicates by enzyme-linked immunosorbent assay (ELISA; ENZO Life Sciences, NY, USA) following the protocol of Carter et al., but with the addition of 500 KIU/µL aprotinin [22, 24–26]. The inter-assay and intra-assay coefficients of variability were reported to be 12.6–13.3 and 11.8–20.9%, respectively [27].

Objective UCs were monitored by cardiotocography. The frequency of subjective UCs was asked from the participants at preinhalation, and at 30 and 60 min postinhalation.

For the secondary objective, the volume of the collected saliva and the number of attempts to collect saliva, both of which constitute saliva collection ability, were recorded. To evaluate whether our intervention protocol can be conducted without causing stress [28], the salivary cortisol level was measured at the same four time points of oxytocin level measurement. Saliva was collected in a separate tube to measure oxytocin and cortisol levels at each measurement point. The assay was conducted by ELISA in duplicates (Salimetrics, PA, USA) following the manufacturer’s instructions.

Negative effects included an abnormal fetal heart rate pattern during the intervention as monitored by cardiotocography, and neonatal outcomes (Apgar score < 7 and neonatal intensive care unit admission) were collected from the medical records. For the assessment of acceptability, the burden of intervention (inhalation, saliva and buccal mucosa collection, and participation) in both groups and the scent perception (preference and strength) in the experiment group were clarified at the end of the intervention.

Some basic characteristics which were reportedly related to a lower oxytocin level were identified [29–34]. The Additional file 3 shows questionnaires and oral questions.

#### Data analysis

Outcomes were descriptively analyzed. The changes between the preinhalation and postinhalation oxytocin and cortisol levels and UC were compared between groups. Additionally, changes in oxytocin levels were statistically compared within groups by the Friedman test and between groups by the Mann–Whitney U test with a two-sided 5% level of significance using SPSS version 25.0J for Windows.

### Results

Eleven women received the intervention: 5 (E1–5) in the experiment group and 6 (C1–6) in the control group. The Additional file 4 shows the participants’ flow chart. Most of the participants’ characteristics were similar between the groups (Table 1).

**Table 1:** Basic characteristics of participants

	Experiment group (n = 5)	Control group (n = 6)	*p* value
Age [SD] (years)	31.4 [2.7]	32.6 [1.8]	0.429
Gestation weeks [SD]	38.2 [0.2]	38.2 [0.1]	0.662
BMI [SD]	23.4 [3.5]	24.6 [2.5]	0.662
Education ≥ 12 years	5	5	1.000
Married	5	6	–
Having children	1	2	1.000
Oxytocin receptor polymorphisms			
rs 53576 (GG)	0	1	1.000
rs2254298 (GG)	3	2	0.567
rs1042778 (TT)	1	0	0.455
Depression (CES-D ≥ 16)	0	0	–
Anxiety			
A-Trait [SD]	31.0 [3.3]	30.0 [3.1]	0.126
A-State [SD]	26.6 [3.4]	32.6 [7.4]	0.177

Oxytocin receptor single nucleotide polymorphism was assayed using buccal mucosa samples collected after completing all saliva collections (TaqMan SNP genotyping assays, Applied Biosystems, Thermo Fisher, MA, USA)

The characteristics reportedly related to a lower oxytocin level were oxytocin receptor polymorphism (GG in rs53576 and rs2254298, and TT in rs1042778) [31–33]; having depression [29, 30, 32], anxiety [33], a high body mass index and no children, and being married [34]. Depression and anxiety were assessed using the Japanese version of CES-D [35, 36] and STAI [37, 38]

*SD* standard deviation, *BMI* Body mass index, *CES-D* Center for Epidemiologic Studies Depression Scale, *A-trait* Trait anxiety of State-Trait Anxiety Inventory, *A-State* state anxiety of State-Trait Anxiety Inventor‎y

*p-value* Mann–Whitney U test or Fisher’s exact test for comparisons between groups

#### Primary outcomes: limited efficacy

**Fig. 1:**
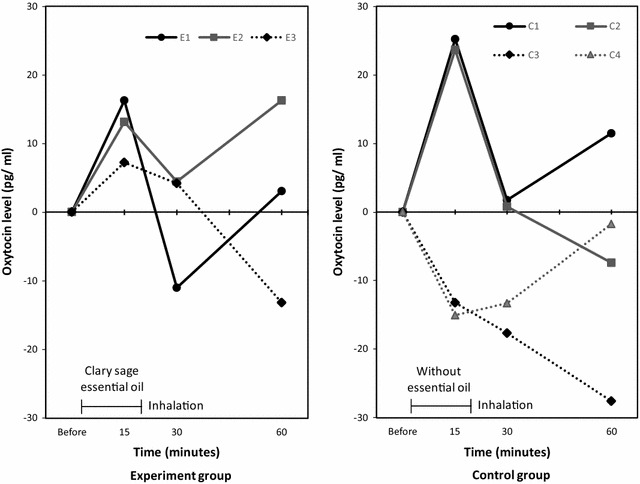
Changes in preinhalation and postinhalation oxytocin levels. Left: Experiment group, n = 5, E4 and E5 are not shown because the oxytocin levels at all the time points were unmeasurable; Right: Control group, n = 6, C5 and C6 are not shown because the oxytocin level at all the time points were unmeasurable. Comparison within groups, no significant differences in the oxytocin levels were found between preinhalation and postinhalation (experiment group: *p* = 0.241; control group: *p* = 0.682). Comparison between groups, no significant differences were found in the preinhalation levels of oxytocin (*p* = 0.310) and the changes between preinhalation and 15 min (*p* = 1.000), 30 min (*p* = 0.190), and 60 min (*p* = 0.690) postinhalation

The oxytocin level could be measured at all the measurement points in 3 women in the experiment group (Fig. 1 E1–3). Their oxytocin levels increased at 15 min and then decreased at 30 min postinhalation. The oxytocin level could be measured at all the measurement points in 4 women in the control group (Fig. 1 C1–4). The oxytocin level at 15 min postinhalation increased in 2 women and decreased in 2 women. However, there were no significant differences in the oxytocin level changes within groups and between groups. Postinhalation objective and subjective UCs were not observed in both groups.

#### Secondary outcomes: practicality

For saliva collection, 3 of the 11 women (27.2%) failed to collect 1.0 mL of saliva even with 4 attempts. The other women could collect 1.0 mL or more of saliva within 3 attempts.

For oxytocin level measurement, 44 saliva samples were collected in both groups. Of the 44 samples, 9 contained 1.5 mL or more of saliva and the oxytocin level could be measured in all of them. Of the 44 saliva samples, 35 contained less than 1.5 mL of saliva and the oxytocin level could not be measured in 10 of these 35 samples (28.5%) owing to the small saliva volume postcentrifugation.

**Fig. 2:**
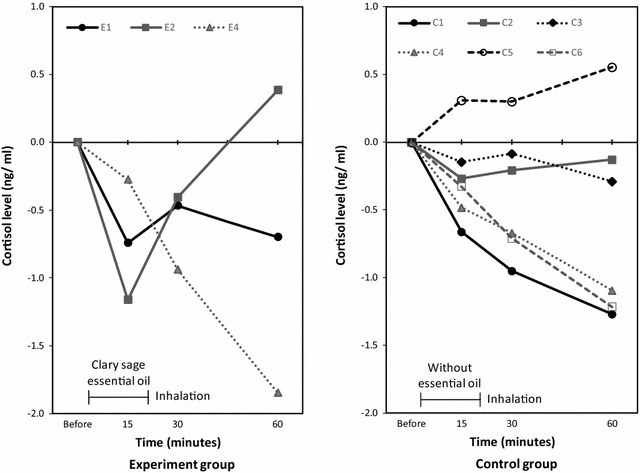
Changes in preinhalation and postinhalation cortisol levels. Left: Experiment group, n = 5, E3 and E5 are not shown because the cortisol levels at all the time points were unmeasurable. Right: Control group, n = 6

The cortisol level at all the measurement points could be measured in 3 women in the experiment group (Fig. 2 E1, E2, E4). All their cortisol levels decreased at 15 min postinhalation. All the postinhalation cortisol levels remained lower than baseline, except in E2. In the control group, the cortisol level at all the measurement points could be measured in all the women (Fig. 2 C1–6). The cortisol levels at all the postinhalation measurement points were lower than baseline, except in C5.

Any negative effects on the fetal heart rate pattern during the intervention and on neonatal outcome were not observed.

#### Secondary outcomes: acceptability

All the burdens of the intervention were judged as moderate or light in both groups. All the women in the experiment group liked the scent of clary sage essential oil. The strength of the scent was judged as appropriate in E1 and E3, and as weak in E2, E4, and E5 in which the oxytocin level could be measured only in E2. The postinhalation oxytocin level in E2 was higher than the baseline.

### Discussion

#### Limited efficacy: oxytocin level and uterine contraction

Inhalation of the scent of clary sage essential oil induced an increasing trend in the oxytocin level at 15 min postinhalation which was measured during the inhalation. This increase, however, could not be maintained at 30 min postinhalation measured at 10 min after finishing the inhalation. Thus, future studies should measure the oxytocin level at 15 min postinhalation and during or immediately after the inhalation of the scent of clary sage essential oil to evaluate the effects.

Uterine contraction was expected to be induced in accordance with the increase in the oxytocin level. However, postinhalation subjective and objective UCs were not observed in the experiment group. Regarding oxytocin receptors, their amount increases around the onset of labor [39] and their polymorphism is related the duration of labor and delivery [40]. Therefore, UC is affected by the oxytocin level as well as the amount and polymorphism of oxytocin receptors. Another possibility is that the increase in the oxytocin level might be small to cause UCs. Clary sage essential oil contains sclareol and is expected to have estrogen-like effects because sclareol has a structure similar to estrogen [41]. As estrogen enhances the release of oxytocin [34], it can be expected that a greater absorption of sclareol into the body would mean a larger increase in the oxytocin level. The present study used inhalation as an absorption route of the essential oil into the body, although skin absorption is also used in aromatherapy. In future studies, administration methods that facilitate the simultaneous skin and inhalation absorption of essential oils such as using a footbath should be adopted for cumulative effects on oxytocin release.

#### Practicality: saliva collection ability, cortisol level, and negative effects

In all the samples with 1.5 mL or more of saliva, the oxytocin level could be measured. Notably, 27.2% of women failed to collect 1.0 mL of saliva even with 4 attempts. The oxytocin level measurement can be further improved by collecting a minimum amount of 1.5 mL of saliva. Moreover, the 27.2% of women with deficient saliva sample should be estimated in the sample size analysis. To obtain adequate saliva volumes, any stimuli that promote salivation consistently at each saliva collection may serve as a solution [14, 42]. Notably, studies indicating the required saliva volumes and deficiencies in the appropriate saliva volume for oxytocin level measurement remain scarce. Recently, the number of studies on oxytocin has been increasing, and novel findings can also be useful for other oxytocin studies.

The cortisol level showed a decreasing trend during the inhalation intervention in both groups, indicating the absence of intervention-induced stress. The intervention protocol had no negative effects.

#### Acceptability: burden of intervention and scent perception

The intervention protocol was within the acceptance level of the participants. Although 3 of 5 women judged the scent as weak, the oxytocin level increased postinhalation. In future studies, the same strength or a stronger scent can be considered.

### Conclusions

This feasibility pilot study showed that inhalation of the scent of clary sage essential oil induced an increasing trend in the oxytocin level, but had no effect on UC. The intervention protocol showed good acceptability. In terms of practicality, it can be further adjusted to enable the collection of a sufficient amount of saliva for optimal measurement of the oxytocin level.

## Limitations

- The limited efficacy of the inhalation could not be clearly confirmed owing to some missing oxytocin values.
- The method of oxytocin measurement did not include an extraction step, which has bearing on the reliability of the measurements. Future studies should consider the inclusion of an extraction step.
- The experiment group could easily guess the presence of the scent of clary sage essential oil which they liked. Thus, an increase in the oxytocin level from the perception of any preferred scent is possible.

## Additional files

**Additional file 1.** Participants’ criteria and request to participants until intervention. Details of the inclusion and exclusion criteria of the participants and requests to the participants to avoid any possible disturbance of the oxytocin and cortisol measurement by enzyme immunosorbent assay until the intervention.

**Additional file 2.** Inhalation intervention. Details of inhalation intervention.

**Additional file 3.** Questionnaires and oral questions. Preinhalation and postinhalation questionnaires and postinhalation oral questions translated from Japanese to English.

**Additional file 4.** Participants’ flow chart. Flow chart of the participants from recruitment to analysis of outcomes.

## Abbreviations

ELISA: enzyme-linked immunosorbent assay
UC: uterine contraction
