# Supplementary material for: Changes in salivary oxytocin after inhalation of clary sage essential oil scent in term-pregnant women: a feasibility pilot study
Source: BMC Res Notes. 2017 Dec 8;10:717. doi: 10.1186/s13104-017-3053-3 (PMC5721455; doi:10.1186/s13104-017-3053-3)
Supplement: Supplementary file 1 — Additional file 1. Participants’ criteria and request to participants until intervention. Details of the inclusion and exclusion criteria of the participants and requests to the participants to avoid any possible disturbance of the oxytocin and cortisol measurement by enzyme immunosorbent assay until the intervention. [file 13104_2017_3053_MOESM1_ESM.docx]

Additional file 1

**Title of data:** Participants’ criteria and request to participants until intervention

**Description of data:**

Details of the inclusion and exclusion criteria of the participants and requests to the participants to avoid any possible disturbance of the oxytocin and cortisol measurement by enzyme immunosorbent assay until the intervention

**The inclusion criteria** were Asian low-risk pregnant women of singleton pregnancies between 38 and 40 gestation weeks before labor onset; planning spontaneous delivery; and can communicate, read, and write in Japanese.

**The exclusion criteria** were < 25 or > 35 years of age; planning labor induction or cesarean section and anesthesia during labor; a medical history; taking medication; alcohol addiction; smoker; allergy to some foods, medicine, plants, and aromatherapy; and breastfeeding.

**The requests to participants until intervention** were to avoid the following: dental treatment (2 days before); alcohol and sexual behavior (1 day before); caffeine intake (12 hours before); and lunch (1 hour before) [14].
