# Supplementary material for: Changes in salivary oxytocin after inhalation of clary sage essential oil scent in term-pregnant women: a feasibility pilot study
Source: BMC Res Notes. 2017 Dec 8;10:717. doi: 10.1186/s13104-017-3053-3 (PMC5721455; doi:10.1186/s13104-017-3053-3)
Supplement: Supplementary file 2 — Additional file 2. Inhalation intervention. Details of inhalation intervention. [file 13104_2017_3053_MOESM2_ESM.docx]

Additional file 2

**Title of data:** **Inhalation intervention**

**Description of data:**

Details of inhalation intervention

The inhalation intervention for each participant was started at 13:00 and lasted for 2 hours in a quiet room. The participants rinsed their mouth with water, answered a questionnaire, drank 100 mL of water, and watched a silent train movie. After 10 minutes from the water intake, the first saliva sample was collected [14] and the inhalation was started.

The clary sage essential oil was diluted 50-fold with 10 mL of odorless propylene glycol in a glass bottle. The bottle was sealed with a silicon cap which had 2 holes with inserted glass tubes. One glass tube was positioned inside the propylene glycol and the opposite side was connected to an air pump (JP-3000, Tokyo Deodorant Co., Tokyo, Japan). The other glass tube was positioned outside the propylene glycol in the bottle and connected to a glass funnel. The funnel was set 10 cm away from the participants’ nostril [16]. The experiment group inhaled the scent produced at a rate of 2.0 L per minute for 20 minutes. Clary sage essential oil (*Salvia sclarea*, Lot No. 57, Tree of life, Tokyo, Japan) contained 55.8% linalyl acetate, 23.5% linalool, 3.3% alpha-terpineol, 2.1% beta-caryophyllene, 2.1% geranyl acetate, 1.1% germacrene D, and 0.6% sclareol [15].
