# Supplementary material for: Changes in salivary oxytocin after inhalation of clary sage essential oil scent in term-pregnant women: a feasibility pilot study
Source: BMC Res Notes. 2017 Dec 8;10:717. doi: 10.1186/s13104-017-3053-3 (PMC5721455; doi:10.1186/s13104-017-3053-3)
Supplement: Supplementary file 3 — Additional file 3. Questionnaires and oral questions. Preinhalation and postinhalation questionnaires and postinhalation oral questions translated from Japanese to English. [file 13104_2017_3053_MOESM3_ESM.docx]

**Thank you for your participation in this study involving the inhalation of the scent of clary sage essential oils today.**

- Please indicate your physical and mental states below the questionnaire. The aim of the questionnaire is to assess any changes in the hormone levels and not to evaluate you and your family.
- This questionnaire has 6 pages including both sides, and requires approximately 10 minutes to complete.

Please read the instructions carefully and fill in the questionnaire.

# **Please answer the questions about your mental state.**

## [Abbreviation, please see the Japanese version of Center for Epidemiologic Studies Depression Scale.]

## [Abbreviation, please see the Japanese version of state anxiety of State-Trait Anxiety Inventory.]

## [Abbreviation, please see the Japanese version of trait anxiety of State-Trait Anxiety Inventory.]

# **Fill in the blanks or encircle the answer. Please describe your current situation if otherwise specified.**

Thank you very much for your answers.

This is all for the questions before the inhalation.

# Thank you for your participation in the inhalation of the scent of clary sage essential oils. Please answer the questions below including some similar questions before the inhalation. This questionnaire has 3 pages including both sides and requires approximately 5 minutes to complete.

# **Did you like the scent you inhaled today? Please put a tick in □.**

- I liked the scent.　　　　　　　　　　　　□ I did not like the scent.

Please go ahead.

# [Abbreviation, please see the Japanese version of state anxiety of State-Trait Anxiety Inventory.]

# [Abbreviation, please see the Japanese version of trait anxiety of State-Trait Anxiety Inventory.]

**That is all for today. Thank you very much!**

# **Postinhalation oral questions.**

Please give your feedback regarding the study protocol.

1. Did you feel burdened with the inhalation of the scent? (i.e., light, moderate, and heavy)
2. Did you feel burdened with the saliva collection? (i.e., light, moderate, and heavy)
3. Did you feel burdened with the buccal mucous membrane collection? (i.e., light, moderate, and heavy)
4. Did you feel burdened with your participation in the intervention procedure? (i.e., light, moderate, and heavy)
5. How was the strength of the scent for you? (only for the experiment group, i.e., weak, moderate, and strong)
