# Supplementary material for: Changes in salivary oxytocin after inhalation of clary sage essential oil scent in term-pregnant women: a feasibility pilot study
Source: BMC Res Notes. 2017 Dec 8;10:717. doi: 10.1186/s13104-017-3053-3 (PMC5721455; doi:10.1186/s13104-017-3053-3)
Supplement: Supplementary file 4 — Additional file 4. Participants’ flow chart. Flow chart of the participants from recruitment to analysis of outcomes. [file 13104_2017_3053_MOESM4_ESM.docx]

Additional file 4

**Title of data:** **Flow chart of participants**

**Description of data:**

Flow chart of the participants from recruitment to analysis of outcomes
